# Comprehensive RNA-Seq-based study and metabolite profiling to identify genes involved in podophyllotoxin biosynthesis in *Linum album* Kotschy ex Boiss. (Linaceae)

**DOI:** 10.1038/s41598-023-36102-7

**Published:** 2023-06-07

**Authors:** Zahra Danaeipour, Ghasemali Garoosi, Masoud Tohidfar, Mohammad Reza Bakhtiarizadeh, Mohammad Hossein Mirjalili

**Affiliations:** 1 Department of Biotechnology, Faculty of Agriculture and Natural Resources, Imam Khomeini International University, Qazvin, 3414916818 Iran; 2 Department of Cell and Molecular Biology, Faculty of Life Sciences and Biotechnology, Shahid Beheshti University, Tehran, 1983969411 Iran; 3 Department of Animal and Poultry Science, College of Aburaihan, University of Tehran, Tehran, 3391653755 Iran; 4 Department of Agriculture, Medicinal Plants and Drugs Research Institute, Shahid Beheshti University, Tehran, 1983969411 Iran

**Keywords:** Biotechnology, Genetics, Plant sciences

## Abstract

*Linum album* is a well-known rich source of anticancer compounds, i.e., podophyllotoxin (PTOX) and other lignans. These compounds play an important role in the plant’s defensive system. The RNA-Seq data of flax (*L. usitatissimum*) were analyzed under various biotic and abiotic stresses to comprehend better the importance of lignans in plant defense responses. Then, the association between the lignan contents and some related gene expressions was experimented with HPLC and qRT-PCR, respectively. Transcriptomic profiling showed a specific expression pattern in different organs, and just the commonly regulated gene *EP3* was detected with a significant increase under all stresses. The in silico analysis of the PTOX biosynthesis pathway identified a list of genes, including laccase (*LAC11*), lactoperoxidase (*POD*), 4-coumarate-CoA ligase (*4CL*), and secoisolariciresinol dehydrogenase (*SDH*). These genes increased significantly under individual stresses. The HPLC analysis showed that the measured lignan contents generally increased under stress. In contrast, a quantitative expression of the genes involved in this pathway using qRT-PCR showed a different pattern that seems to contribute to regulating PTOX content in response to stress. Identified modifications of critical genes related to PTOX biosynthesis in response to multiple stresses can provide a baseline for improving PTOX content in *L. album*.

## Introduction

The genus *Linum* (Linaceae family) comprises about 230 species, including *L. album* Kotschy ex Boiss., *L. flavum* L*.*, and *L. mucronatum* Bertol that are known as valuable sources of lignans, especially podophyllotoxin (PTOX)^1–3^. However, the production and accumulation pattern of lignans may vary from one species to another^4^. Lignans have broad applications in traditional and modern medicine and the food industry. Due to the cytotoxicity of PTOX, its semi-synthetic derivatives, i.e.*,* Etoposide and Teniposide with anticancer activities, are currently produced in the pharmaceutical industry^5,6^.

**Figure 1:**
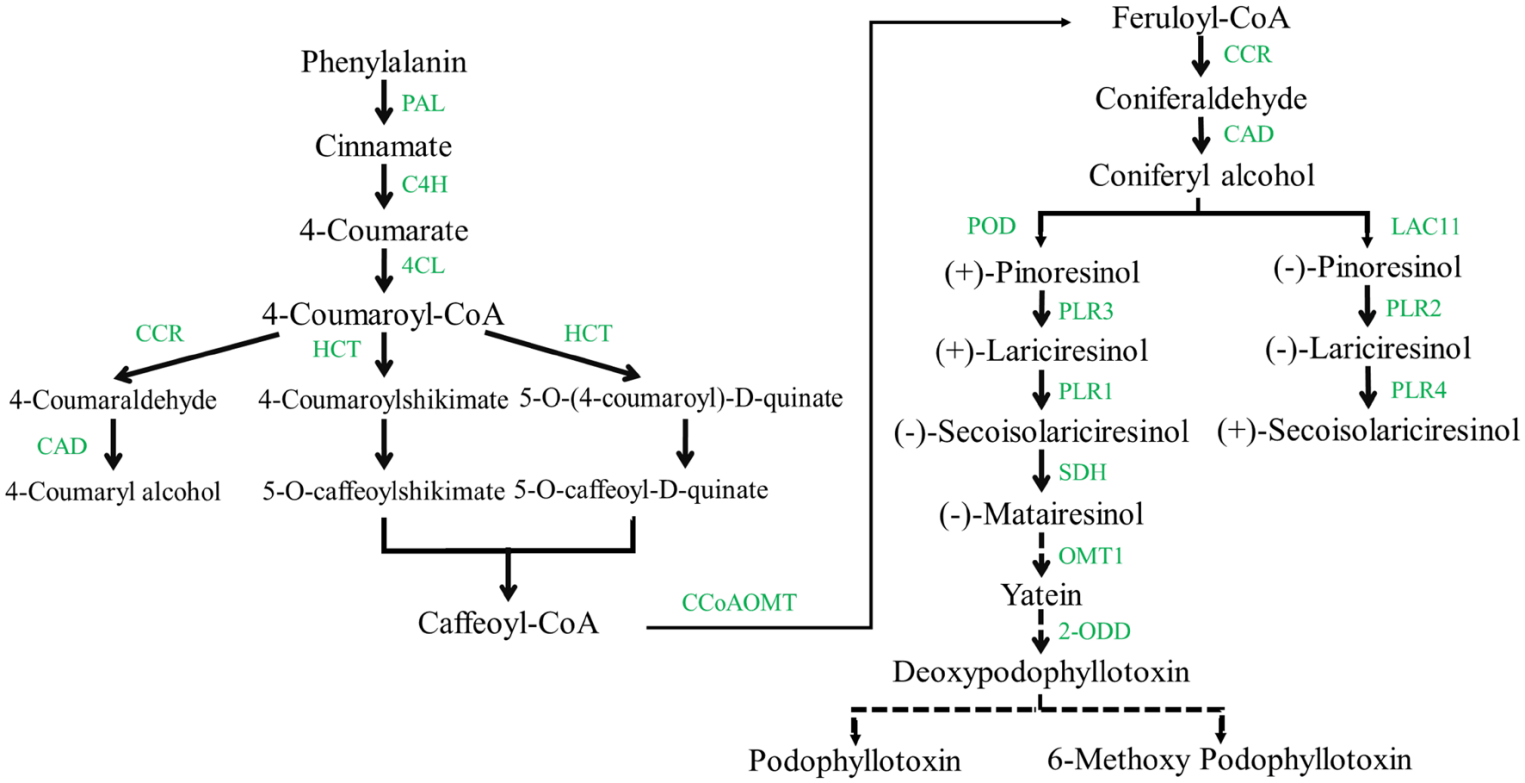
Putative biosynthetic pathway of podophyllotoxin (obtained by KEGG, http://www.kegg.jp/kegg/kegg1.html, and Phytozome, https://phytozome.jgi.doe.gov, database). The dashed arrow indicates several uncharacterized steps. Enzyme abbreviations are as follows: PAL, Phenylalanine ammonia lyase; C4H, Cinnamate 4-hydroxylase; 4CL, 4-coumarate-CoA ligase; HCT, Shikimate O-hydroxycinnamoyltransferase; CCoAOMT, *trans*-caffeoyl-CoA 3-O-methyltransferase; CCR, cinnamoyl-CoA reductase; CAD, Coniferyl-alcohol dehydrogenase; POD, Lactoperoxidase; LAC11, Laccase; PLR, Pinoresinol/lariciresinol reductase; SDH, Secoisolariciresinol dehydrogenase; OMT1, 5′-desmethyl-yatein O-methyltransferase; 2-ODD, Deoxypodophyllotoxin synthase.

PTOX and its derivative compounds, particularly 6-methoxypodophyllotoxin (6-MPTOX), are the major aryltetralin lignans resulting from the shikimic acid/phenylpropanoid pathway^7^ (Fig. 1). In the phenylpropanoid pathway, the matairesinol is generated by secoisolariciresinol (SECO) oxidation through SECO dehydrogenase (SDH). The role of lignans has been reported in plant defense responses against abiotic and biotic stresses^5^.

Expression of some genes encoding enzymes in the biosynthetic pathway of PTOX was recognized in *L. flavum*, *Forsythia koreana* Rehder Nakai and *Podophyllum hexandrum* Royle^8^. However, downstream genes have not yet been completely elucidated in the *L. album*^9^. Hence, transcriptome and metabolome profiling is necessary to explore gene expression patterns of PTOX biosynthesis in *L. album* to improve the production of this valuable anticancer compound.

Gene expression studies increasingly rely on high-throughput sequencing (RNA-Seq), while they are usually performed on a limited number of biological replicates and experimental conditions due to the high cost of RNA-Seq. Therefore, the desired results can be explored by integrating and analyzing the datasets. Several RNA-Seq studies have been reported on genes associated with an individual’s response to stress and textile properties in the flax. For example, changes in the gene expression have been previously evaluated using RNA-Seq sequencing in flax genotypes with different tolerance under aluminum (Al) toxicity, drought stress, *Fusarium* treatment, and zinc (Zn) deficiency^10–13^. Gene expression changes were unique and specialized for each stimulus. Under aluminum toxicity, NAC (NAM, ATAF, and CUC) transcription factors (TFs), along with MADS-box (for MCM1, AG, DEFA, and SRF), regulated plant growth and led to Al tolerance by interfering with cell wall alterations^13^. However, under drought stress, gene expression patterns changed to cope with water deficit toward proline biosynthesis and DNA repair, indicating the important role of proline in drought tolerance of flax^10^.

Drought stress is one of the major limiting factors. This stress can affect the growth and geographical distribution of the plants worldwide, followed by a plenty reduction in crop yields. Severe climate change increases the frequency of harsh drought conditions leading to multiple stresses such as drought and heat or drought and K^+^ deficiency stress^14,15^. Through evolution, plants adapt themselves to elude the lethal effects of stresses via the favorable physiological, biochemical, and molecular properties as well as through transcriptional and epigenetic regulations^16^.

*L. usitatissimum* and *L. album* grow in arid and semi-arid regions of Iran. Thus, they are often exposed to drought and other stresses in their natural habitat^17^. It can be concluded that both species have good resistance to the stresses mentioned above, but the commonly regulated responsive genes of the plants have not been investigated using transcriptome studies under multiple stresses.

The present work conducted a comprehensive RNA-Seq analysis of published datasets on *L. usitatissimum,* a model plant containing lignans, using the same pipeline. This approach would let us find common and unique transcriptional changes at the genome-wide level, particularly changes associated with lignans biosynthesis occurring during short-term abiotic and biotic stresses^10–13,18^. The present study aimed to focus on the changes in the gene expression patterns involved at the main core of the PTOX biosynthesis pathway, which can be directly affected by different stresses and limiting factors, i.e., drought and K^+^ deficiency. These findings can be interestingly considered to better explain the molecular basis of PTOX biosynthesis in the genetic engineering or genome-editing approaches for the improved PTOX production.

## Results

### RNA-Seq data and transcriptome responses

A total of 1,358,545,065 pair- and single-end raw reads were obtained from six RNA-Seq datasets, ranging from 18.15 to 90.14 million per sample. Among these, 275,217,314, 130,295,728, 109,809,110, 402,917,502, 220,197,810 and 220,107,601 million reads belonged to drought, ABA, K^+^ deficiency, *Fusarium*, Al toxicity and Zn deficiency treatments, respectively. After trimming, 10% of the reads were removed, giving the quality of the datasets. The clean reads with an average of 94.13% read mapping rate were aligned to the *L. usitatissimum* genome (Table 1).

**Table 1:** Overview transcriptome data used in this study.

Stress	No. Sample	Cultivar	Sample tissue	Time afer stress	Replicatesper sample	Accession	Number of reads	mapping rate (%)
Drought stress	12	Z141	Leaf	20 day	3	PRJNA598287	275,217,314	94.72
		NY17						
ABA	2	NIKE	Plant	14 day	–	PRJNA515812	130,295,728	92.71
K^+^deficiency	12	Sofie	Plant	21 day	3	PRJNA414507	109,809,110	96.31
Fusarium	8	Regina	Plant	14 day	3	PRJNA504749	402,917,502	92.99
		Nike						
Al toxicity	8	TMP1919-N Hermes	Root	7 day	2	PRJNA343117	220,197,810	92.36
Zn deficiency	6	Norlin	Root	21 day	4	PRJNA497472	220,107,601	94.96

**Figure 2:**
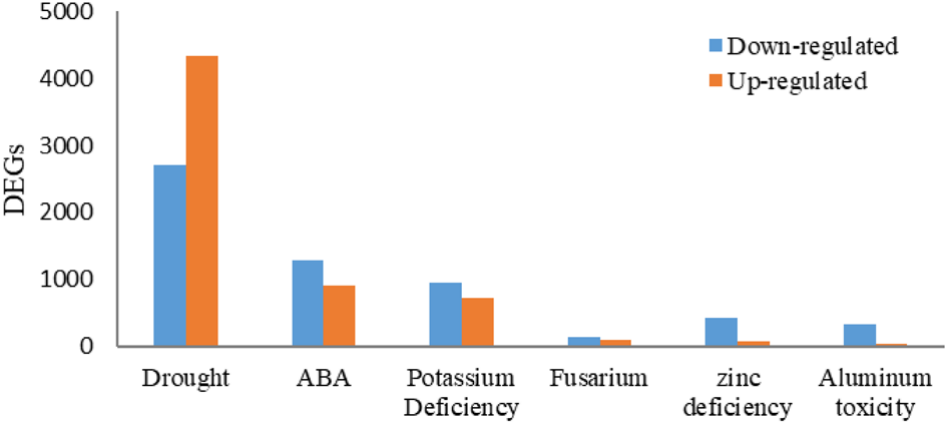
DEGs identified under abiotic and biotic stresses in *L. usitatissimum.*

The results from RNA-Seq analysis showed that the total number of differentially expressed genes (DEGs) was in the range of 314 (*Fusarium* wilt) to 15,659 (drought stress), depending on stress treatment. In drought stress, the number of upregulated DEGs (4328) was more than that of downregulated DEGs (2709). While in other stresses, the number of downregulated genes was higher (Fig. 2). A large number of DEGs indicated that a considerable portion of the transcriptome was engaged in combating drought stress.

**Figure 3:**
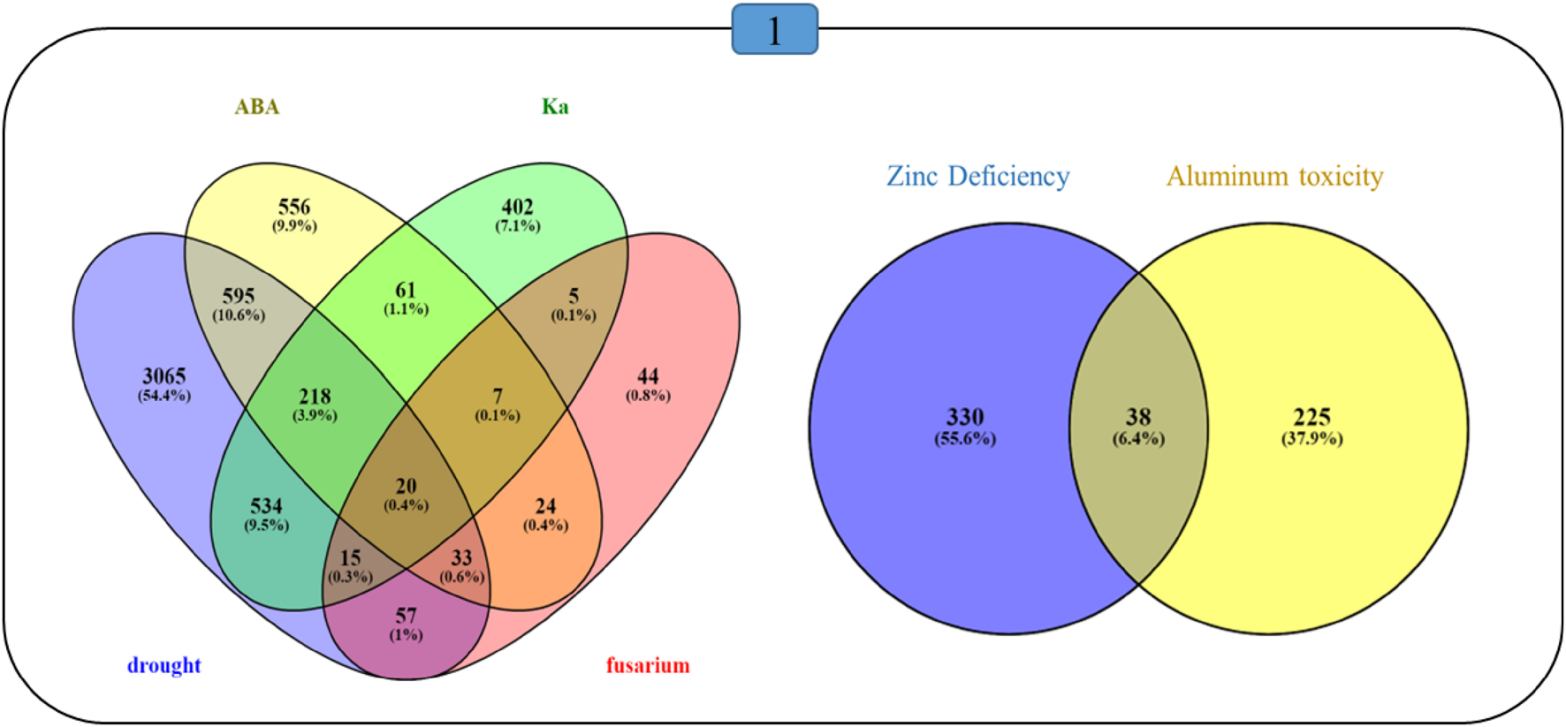
Comparison between transcriptomic responses to abiotic and biotic stresses in *L. usitatissimum* venn-diagram showing the number of commonly regulated and unique genes responsive to drought, ABA, K^+^ deficiency, *Fusarium* treatment (left), Al toxicity and Zn deficiency (right). The blue square (top) indicates the commonality between all treatments.

The common genes among the datasets were revealed using the overlapping areas in the Venn diagrams (Fig. 3). The overlapping areas shared the 238 commonly regulated genes responsive to abiotic stresses and the 20 commonly regulated genes responsive to both biotic and abiotic stresses in the shoot. Also, the 38 commonly regulated genes responsive to abiotic stresses were observed in the root. Only one gene, AT3G54420, showed a significant change in gene expression between shoot and root among all the studied stresses. AT3G54420 encodes an *EP3* chitinase that catalyzes the hydrolysis of chitin and involves in response to fungal infections and some of the abiotic stresses. Also, 787 commonly regulated genes were obtained from drought stress and K^+^ deficiency data, and this study experimentally examined both stresses.

**Figure 4:**
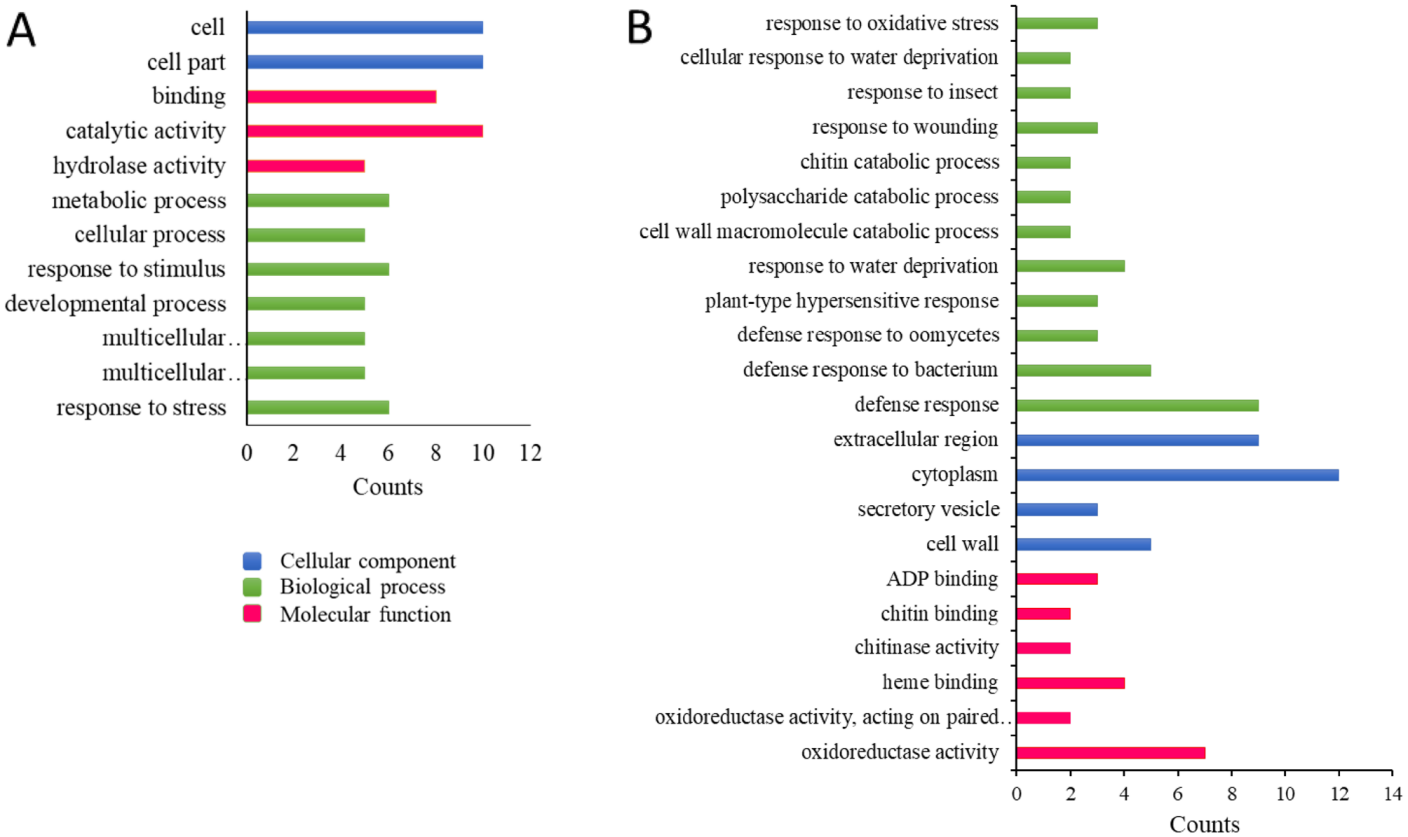
Gene function prediction by gene ontology terms in *L. usitatissimum*; red: Molecular function; green: Biological process; blue: Cellular component. (**A**): shoot; (**B**): root.

A gene ontology (GO) analysis was performed on all commonly regulated genes in the shoot (20 genes) and root (38 genes) to predict their functions. The commonly regulated genes were grouped into three main GO categories, including biological processes, cellular components, and molecular functions (Fig. 4).

The present work focused on the GO terms category of biological processes to explore the similarities and differences in the stress responses in different organs. The GO terms ‘response to stress (GO:0006950)’, ‘response to stimulus (GO:0050896)’, ‘cellular process (GO:0009987)’, and ‘metabolic process (GO:0008152)’ were all enriched within both organs, which indicates the metabolically active state of the cell during reprogramming in response to stress (Fig. 4). Functional classification of the 20 specific commonly regulated genes showed two stress-responsive GO terms in the shoots. In these GO terms, there are three genes encoding transcripts that belong to apetala2/ethylene responsive factor (AP2/ERF, *DRE2B*), homeobox (HB-7, *ATHB-7*), and NAC (*anac047*) TFs families that significantly upregulated in ABA treatment and drought stress, and downregulated *Fusarium* wilt and K^+^ deficiency. These TFs are known as the main regulators of adverse resistance pathways. The subtilisin-like serine protease (*ARA12* and *SDD1*) genes, encoding a serine-type endopeptidase, showed the highest upregulation (respectively, 7.88 and 7.21), while the lowest downregulation (− 6.10) was observed in the myo-inositol oxygenase 2 (*MIOX2*) gene under the fusarium stress. Therefore, the genes related to common GO terms showed different expression behaviors under individual stressors in an organ. Interestingly, up- or downregulation is directed towards the adjustment cell wall.

Out of 12 functional categories of 38 specific commonly regulated genes in the roots, 8 GO terms exhibited significant enrichments in response to stress, including defense response to a bacterium, insect, oomycetes, water deprivation, wounding, oxidative stress, and plant-type hypersensitive response. These GO showed the highest upregulation for AT1G06620 (3.8) under Al toxicity stress, whereas most genes were downregulated. The lowest amount of downregulation (− 9.22) was observed in AT2G43590 under the Zn deficiency stress. In fact, these individual stresses may cause to produce different adaptive responses, which sometimes have different components; some were upregulated in one instance of individual stress while downregulated in another.

For a better insight into the functions and the metabolic roles, DEGs were analyzed through MapMan. The commonly regulated genes related to shoot and root in six stresses were common to the eight MapMan bincode classifications. The commonly regulated genes were specifically altered within minor CHO metabolism and RNA regulation of transcription in the shoot (Table 2). Whereas commonly regulated genes were specifically altered within the processes of TC/org transformation, redox, cell, transport, hormone metabolism, and signaling receptor kinases in the root (Table 3). The MapMan results showed that commonly regulated genes shared the same trend of regulation in ABA treatment and drought stress. As well as, the same trend of regulation obtained in K^+^ deficiency and fusarium wilt while drought stress and K^+^ deficiency had an almost opposite trend. The GO terms of commonly regulated genes were highly grouped in metabolic process, cellular process, and response to stress (31 GO terms) under both drought stress and K^+^ deficiency. A large number of GO terms in response to stress indicate complex regulatory mechanisms controlling gene expression in the face of stress. In metabolic processes, secondary metabolic pathways have a specialized role in responding to stress. This classification observed that 32 genes are involved in specialized metabolites (SMs) biosynthesis, including phenylpropanoids, phenolic compounds, terpenoids, etc.

**Table 2:** DEGs identified in the studies analyzed of shoot (universal DEGs).

BinCode	BinName	fusarium	ABA	k	drought
3	Minor CHO metabolism	Up	Down	Up	Down
16	Secondary metabolism	Down	Up	Down	Up
20	Stress	Up	Down	Up	Up
26	Misc	down/up	Down/up	down/up	Up
27	RNA.regulation of transcription	Up	Down	Up	Down
33	Development	Up	Down	Up	Down
35	Not assigned	Up	Down/up	Up	Down/up
10	Cell wall	Down	Up	Down	Up
11	Lipid metabolism	Up	Down	Down	Down
29.5	Protein.degradation	Down/up	Up	Down/up	down/up

Up: Gene sets belonging to the corresponding group were upregulation.

Down: Gene sets belonging to the corresponding group were downregulation.

Down/up: Gene sets belonging to the corresponding group were up/downregulation.

**Table 3:** DEGs identified in the studies analyzed of root (universal DEGs).

BinCode	BinName	Zn deficiency	Al toxicity
8	TCA/org. transformation	Down	Down
16	Secondary metabolism	Down/up	Down
20	Stress	Down	Down
21	Redox	Up	Up
26	Misc	Down/up	Down/up
31	Cell	Down	Down
33	Development	Down	Down/up
34	Transport	Down	Down
35	Not assigned	Down	Down
10	Cell wall	Down	Down
11	Lipid metabolism	Down	Down/up
17	Hormone metabolism	Up	Down
29.5	Protein.degradation	Down	Down
30.2	Signalling.receptor kinases	Down	Down

Up: Gene sets belonging to the corresponding group were upregulation.

Down: Gene sets belonging to the corresponding group were downregulation.

Down/up: Gene sets belonging to the corresponding group were up/downregulation.

### Overview transcriptome profiling of SMs

**Figure 5:**
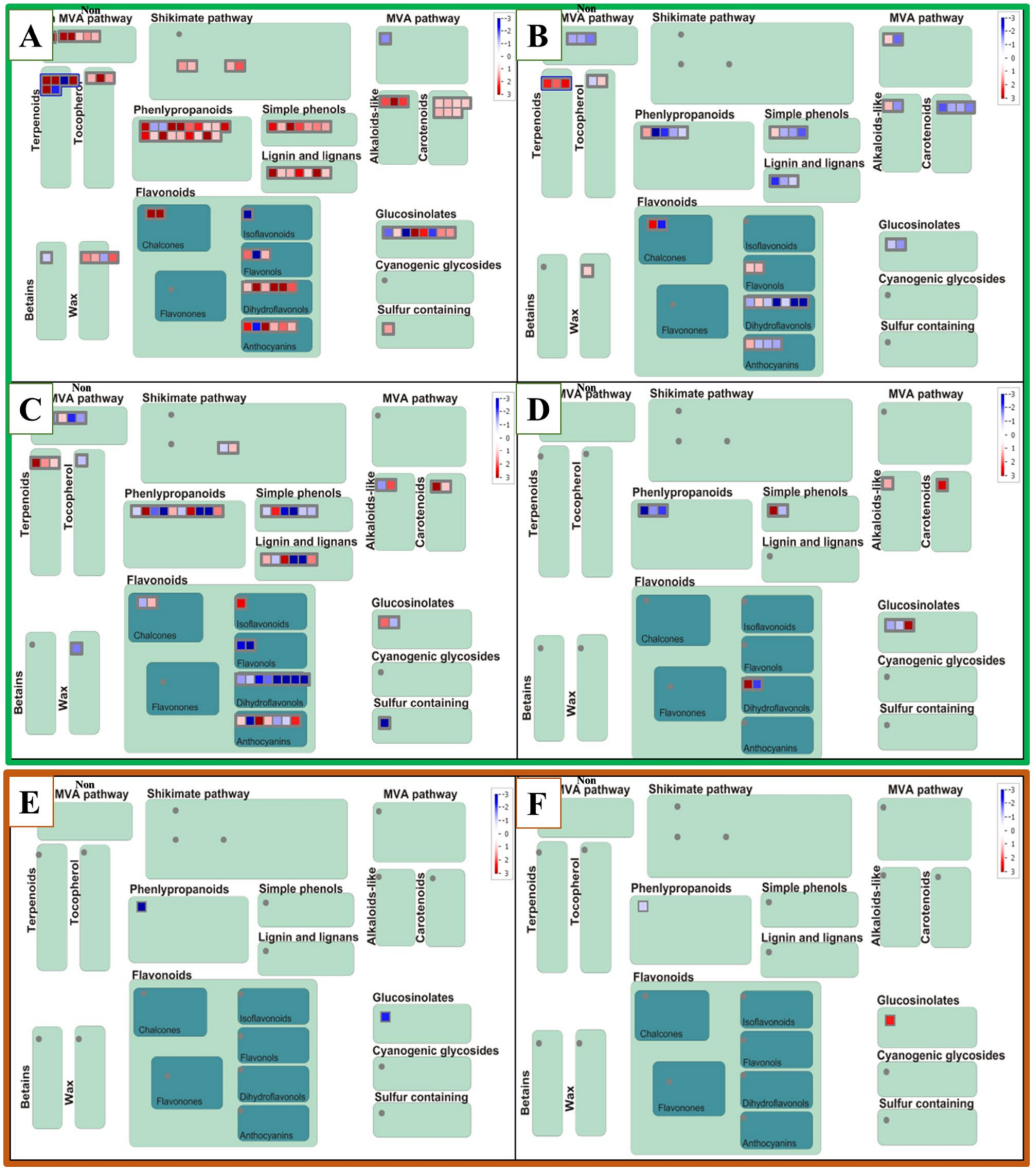
Overview of DEGs of *Linum* species involved in the biosynthesis pathways of specialized metabolites under different stresses. (**A**) drought stress, (**B**) K^+^deficiency stress, (**C**) ABA treatment, (**D**) *Fusarium* treatment, (**E**) Zn deficiency, and (**F**) Al toxicity. Red represented upregulated genes, and Blue downregulated genes.

This study investigated transcript abundance in SMs biosynthetic pathways in flax as a model plant for lignan (using homologous genes analysis with MapMan). Among all the SMs' biosynthetic pathways, phenylpropanoid and flavonoid pathways were highly affected by stresses. The phenolic compounds such as flavonoids and some precursors of lignans are beneficial antioxidants that may be triggered by the plant when it is sensed a stress-specific signal. These compounds are also involved in scavenging ROS produced by secondary oxidative stress resulting from multiple stresses. The type of stress may have a significant effect on specific gene expressions^19^. The highest increase in transcript abundance was *AT4G12300* (7.6-fold) and *AT5G47950* (7.26-fold) for flavonoids and phenylpropanoids under drought stress, respectively (Fig. 5A). The K^+^ deficiency increased 2.5-fold transcript abundance for *AT4G16740* and *AT1G59960* genes involved in the biosynthetic pathways of terpenoids and flavonoids, respectively (Fig. 5B). The ABA treatment also caused an increase of 6.7-fold (*AT5G49555*) and 4.5-fold (*AT4G35160*) in biosynthetic pathways of carotenoids and phenylpropanoids (Fig. 5C). *Fusarium* treatment induced a 3.8-fold increase of AT5G05390 in the biosynthetic pathways of simple phenols (Fig. 5D).

The highest decreased levels were observed in the biosynthetic pathways of glucosinolates (− 5.4 for *AT4G39950*), flavonoids (− 4.15 for *AT5G54010*), and phenylpropanoids (− 3.36 for *AT4G35160*), sulfur-containing. misc.alliinase (− 7.3 for *AT1G70560*), phenylpropanoids (− 3.4 for *AT3G50280*) under drought, K^+^ deficiency, ABA, and *Fusarium* treatment in *L. usitatissimum*, respectively. Roots subjected to stressful conditions showed little changes in the gene expression of SMs (Fig. 5E, F). However, most alterations related to the *AT2G19070* gene in phenylpropanoid biosynthetic pathways.

### Modulating lignans biosynthetic pathway genes during stress exposure

**Figure 6:**
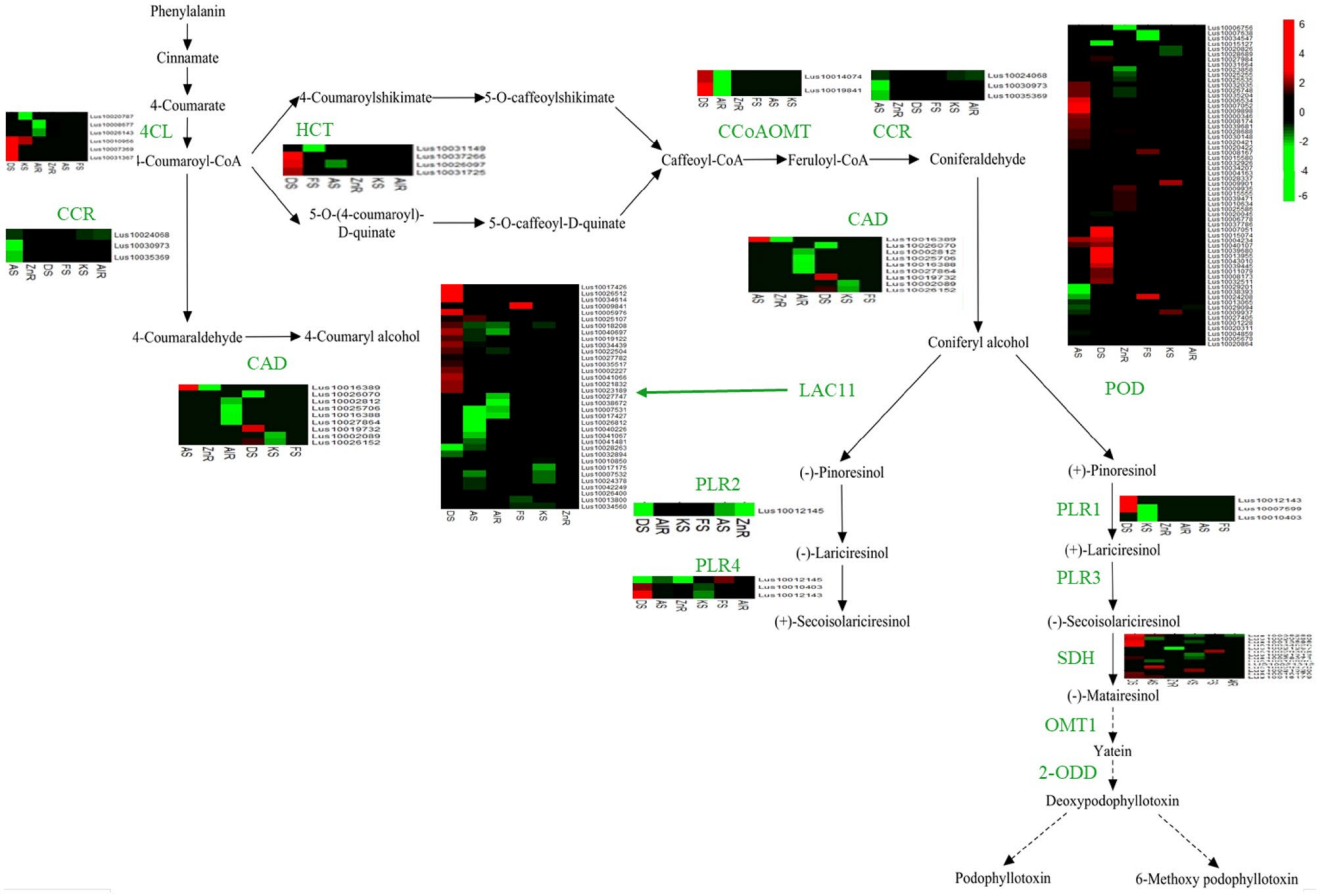
Transcription profiling of genes in podophyllotoxin biosynthesis pathway originating from phenylalanine. The transcriptome data were analyzed under different stress and genes affected in the podophyllotoxin biosynthesis are demonstrated at the studied stresses: DS, Drought-shoot; AS, ABA-shoot; KS, K^+^ deficiency-shoot; FS, Fusarium-shoot; ZnR, Zn deficiency-root; AlR, Al toxicity-root. Expression levels of genes encoding enzymes related to the podophyllotoxin biosynthetic pathway are represented; Red: upregulated; Green: downregulated.

The mining of transcriptome data of the *L*. *usitatissimum,* obtained from shoots and roots, was used to identify various genes implicated in the biosynthesis of PTOX. In this regard, 12 transcripts encoding enzymes of the PTOX biosynthetic pathway were found in *L. usitatissimum*, of which 11 transcripts revealed significant expression in at least one studied stress. Coniferyl alcohol has been known as a critical precursor in PTOX biosynthesis, so earlier and later its biosynthetic steps were considered as upstream and downstream, respectively. The transcripts encoding enzymes, namely 4-coumarate-CoA ligase (4CL), shikimate O-hydroxycinnamoyltransferase (HCT), *trans*-caffeoyl-CoA 3-O-methyltransferase (CCoAOMT), cinnamoyl-CoA reductase (CCR) and coniferyl-alcohol dehydrogenase (CAD), were all upstream. The highest number of upstream transcripts was observed in CAD. Transcripts encoding enzymes, namely LAC11, pinoresinol/lariciresinol reductase (PLR1-4), lactoperoxidase (POD), and SDH, were downstream. The highest increased expression was 6.2, 5.92, 5.3, and 3.93-fold changes for LAC11, POD, 4CL, and SDH under drought stress, respectively (Fig. 6). Interestingly, gene expressions encoding these enzymes were only downregulated under Al toxicity treatment. Therefore, the heatmap results illustrated that drought stress highly affected enzymes of the PTOX biosynthetic pathway. POD and SDH from downstream and 4CL from upstream were positively adjusted, leading to the pathway progressing toward PTOX biosynthesis in the shoot.

**Figure 7:**
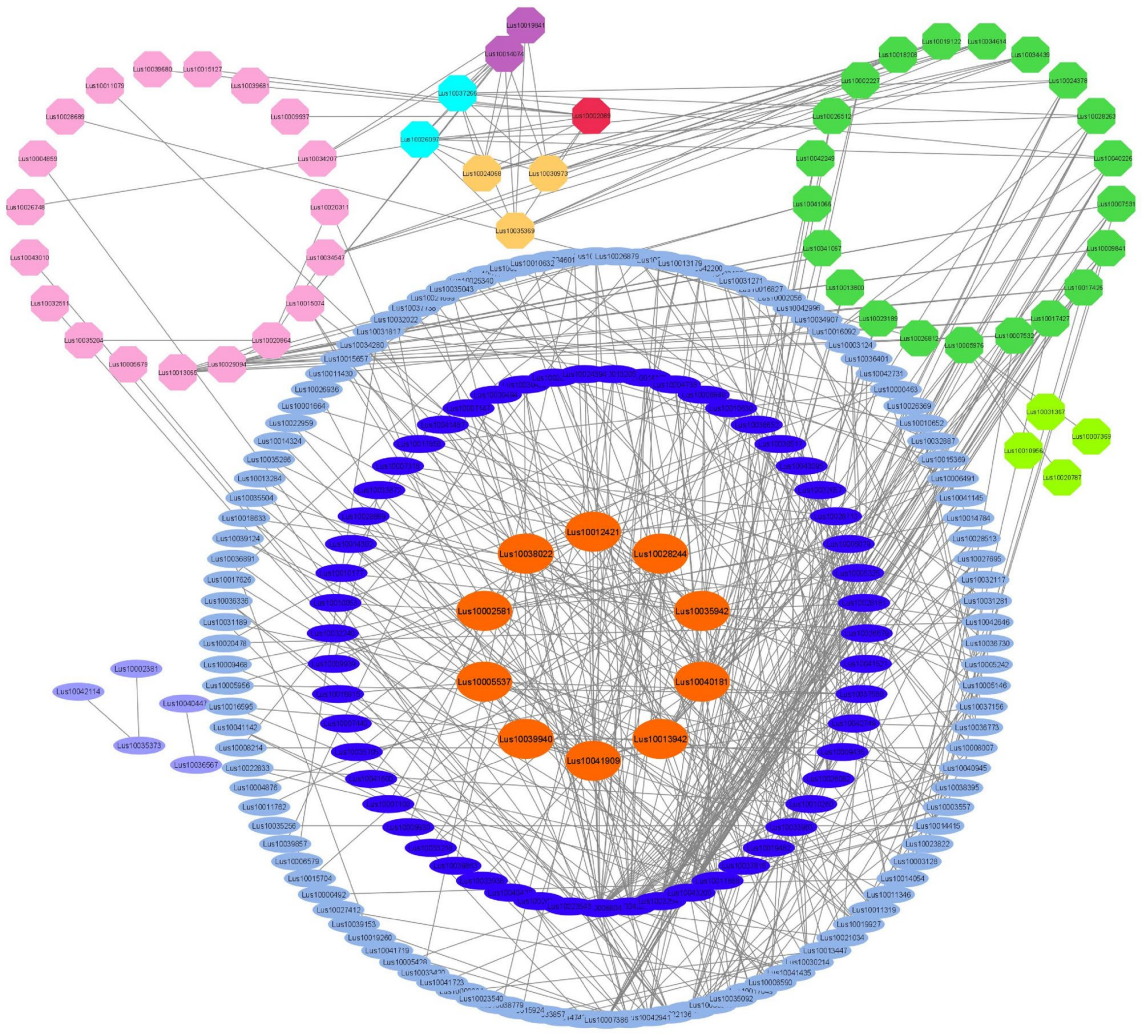
Interaction networks of ID transcriptions in the biosynthetic pathway of podophyllotoxin. ID transcriptions showed a significant difference in at least one stress. Transcription IDs that did not connect to any node were removed and displayed ten hub genes and the first neighbor. Green: LAC11, pink: POD, red: CAD, orange: CCR, dark orange: TFs, light green: 4CL, blue:HCT, purple: CCoAOMT.

To explore the protein interactions, Fisher’s test was performed to find TFs which possess significantly over-represented targets in the transcripts encoding enzymes. Subsequently, a protein–protein interaction (PPI) network constructed for all transcripts encoding enzymes and TFs. Hub genes were defined by the maximum neighborhood component (MNC) ranking algorithm, which obtained higher utilization. PPI network analysis indicated that TFs with the strongest interaction, namely NAC, ARF (auxin response factors), HB, MADS, MYB (myeloblastosis), and AS2, were hub genes. It was then selected first neighbors including AP2/ERF, MYB, MADS, CPP (cystein-rich polycomb-like protein), WRKY, ZF_HD (zinc finger homeodomain), HSF (heat stress transcription factor), ARF, C2H2ZnF (C2H2 zinc-finger), AS2 (asymmetric leaves2), NAC, TCP (TB1, CYC and PCFs), B3, Jmj (JUMONJI), GARP (golden2, ARR-B, Psr1), BBR_BPC (barley b-recombinant/basic pentacysteine), SBP (SQUAMOSA promoter binding proteins), bZIP (basic leucine zipper domain), YABBY, DOF (DNA binding with one finger), and HD. In this network analysis, LAC11 had more interactions with hub TFs, whereas CCoAOMT showed more interactions with other transcripts encoding enzymes (Fig. 7)  (See additional file 2).

### Gene expression changes in response to multiple stresses in *L. album*

**Figure 8:**
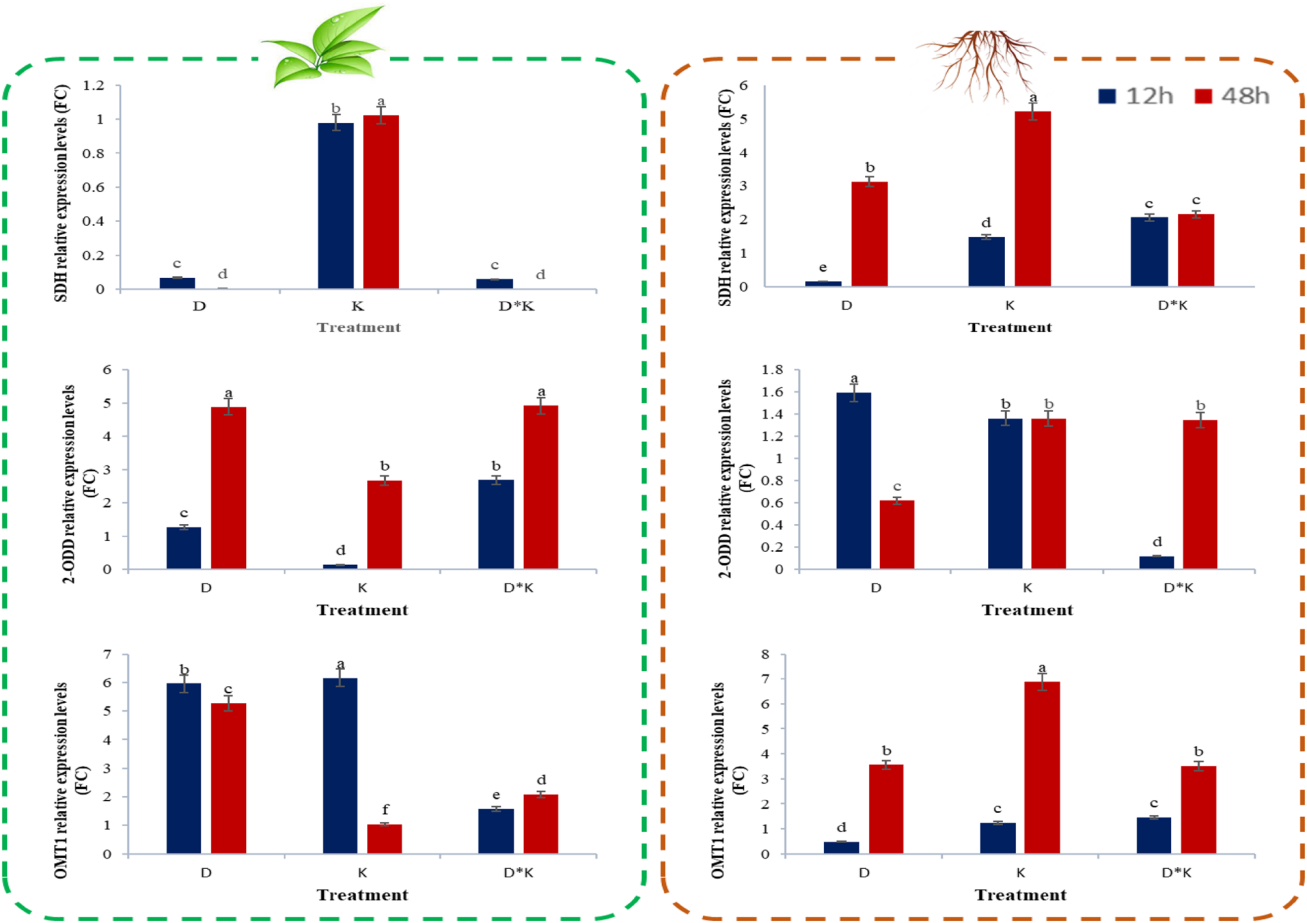
qRT-PCR analysis of the genes involved podophyllotoxin biosynthesis pathway in *L. album.* The *GAPDH* gene was used as an internal control to normalize the expression data. The relative expression levels of each genes were expressed as the fold change between control and other treatment. SDH, Secoisolariciresinol dehydrogenase; 2-ODD, Deoxypodophyllotoxin synthase; OMT1, 5′-desmethyl-yatein O-methyltransferase. The treatments are shown as D, drought; K, K^+^ deficiency; D*K, drought*K^+^ deficiency. Data are expressed as Mean ± standard deviation. Bars not sharing a common letter are significantly different (*P* ≤ 0.01).

To the best of our knowledge, the end of the PTOX biosynthetic pathway is not fully identified in *L. album*, while several genes were identified in the *Podophyllum* species^20^. On the other side, the genes encoding enzyme SDH, which is involved in the branching of downstream lignans of the PTOX biosynthesis pathway, and converts the SECO to matairesinol, were shown an increase in RNA-Seq analyses associated with the shoots of *L. usitatissimum* (Fig. 6). Therefore, the qRT-PCR analysis of *SDH*, 5′-desmethyl-yatein O-methyltransferase (*OMT1*), and deoxypodophyllotoxin synthase (*2-ODD*) genes from downstream was performed to explore the expression patterns under drought stress, K^+^ deficiency, and a combination of both conditions in shoot and root of *L. album*. The relative expression for these genes ranged from 0.00001 to 6.96, with the lowest fold change being for the *SDH* in shoot under combined stress and the highest for the *OMT1* in root under K^+^ deficiency after 48 h treatment. The results showed an increasing trend in *OMT1* for all samples, especially at 48 h subjected to treatment, and *2-ODD* for the shoot samples. At the same time, *2-ODD* decreased under drought stress and had no significant difference under K^+^ deficiency compared with untreated samples. *SDH* exhibited a decreasing trend in all shoot samples except samples under K^+^ deficiency. Simultaneously, all root samples showed an increasing trend (Fig. 8).

### Lignans content changes in response to multiple stresses in the *L. album*

**Figure 9:**
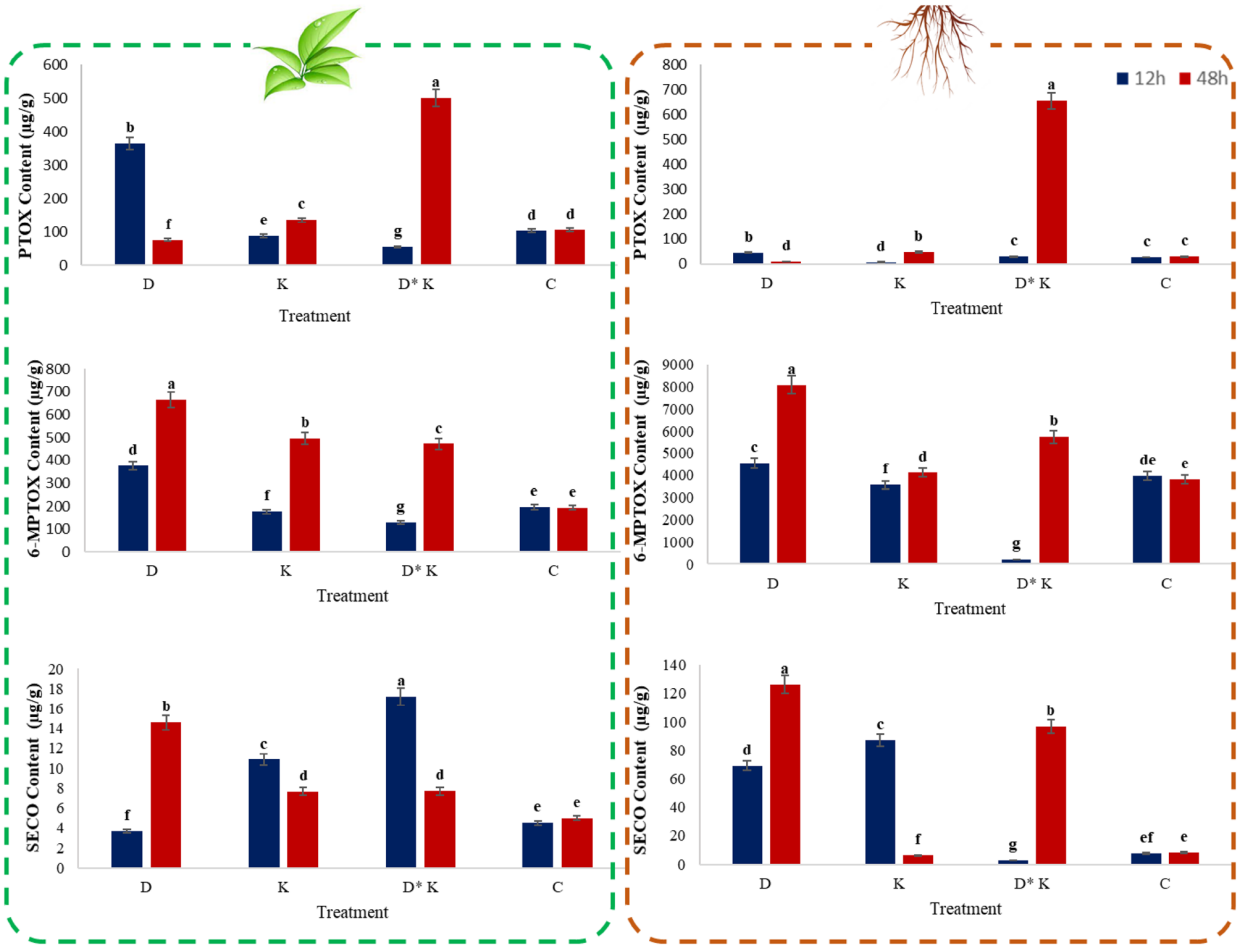
HPLC analysis of lignan contents in *L. album*. The podophyllotoxin, 6-methoxypodophyllotoxin, and secoisolariciresinol content in shoots and roots of *L. album* at 12 and 48 h. PTOX, Podophyllotoxin; 6-MPTOX, 6-methoxypodophyllotoxin; SECO, Secoisolariciresinol. The treatments are shown as D, drought; K, K^+^ deficiency; D*K, drought*K^+^ deficiency and C, control. Data are expressed as mean ± standard deviation. Bars not sharing a common letter are significantly different (*P* ≤ 0.01).

SMs profile corresponded with transcriptome results, and an increasing trend in lignans mostly showed under stress. The 6-MPTOX content was more than PTOX in root and shoot organs. The highest PTOX content (500.25 ± 3.6 µg g^−1^) was in the samples at 48 h subjected to the combination of both conditions, likely due to increasing SECO content and upregulation of *SDH*, *OMT1*, and *2-ODD* genes. The 6-MPTOX content significantly increased in all samples at 48 h compared with untreated samples. The highest values of 6-MPTOX were 664.7 ± 0.64 and 8077.6 ± 152.75 µg g^−1^ in shoot and root under drought stress, respectively. In comparison, the PTOX content of samples subjected to drought decreased after 48 h initial increases. The PTOX content decreased at the roots coincided with a decrease in *2-ODD* expression, whereas 6-MPTOX obtained the highest value. The highest values of SECO were 126.25 ± 0.7 µg g^−1^ and 14.6 ± 0.15 µg g^−1^ in the root and shoot subjected to drought stress, respectively. Due to downregulation of *SDH* and *OMT1* gene expressions, a copious amount of SECO simultaneously accumulated in the roots. SECO content exhibited an initial increase in shoot samples subjected to K^+^ deficiency and the combination of both conditions. According to the duration of treatment, SECO decreased; however, it was more than in the untreated samples (Fig. 9).

## Discussion

The *L. album* is a well-known source of lignans. Several studies have been carried out on their physiological and biochemical properties and expression of some key genes involved in the biosynthesis of lignans in vitro cultures techniques^3,9,21^. This study aimed to recognize a common base of transcriptional changes, especially changes associated with PTOX biosynthesis occurring in different organs during multiple stresses in vivo cultures.

The expression profiles showed obviously differences in the number of up- and down-regulated DEGs under multiple stresses. Fewer DEGs were observed in this plant's stresses related to the roots (such as Al toxicity) compared to the shoots. These differences are driven by differences between datasets that can have various sources, such as biological or technical variability.

Identification of a large number of DEGs under drought stress indicated that a considerable portion of the transcriptome was engaged by this stress^22^. Under drought stress, the differences between tolerance and susceptible genotypes arise in gene expression patterns, and phenotypes^10^. Also, it has been observed that the expression level of stress genes responsive is usually higher in tolerance genotypes^13^. Therefore, these results corroborate the concept that flax has better drought tolerance than other stresses^10^.

According to the identified DEGs, 20 commonly regulated genes responsive to biotic and abiotic stresses and 38 commonly regulated genes responsive to abiotic stresses were overlapped using Venn diagram analyses in the shoot and root, respectively. Then, only one commonly regulated gene, endochitinase *EP3*, was identified with a significant increase in both organs. The *EP3*, catalyzing the hydrolysis of chitin, is involved in the plant development processes, generation of signal molecules, plant defense responses, and programmed cell death^23,24^. The expression of the *EP3* gene increased in response to drought, salinity, treatments of UV light, exogenous elicitor treatment, wounding, and pathogen attack^11,23,24^*.* Moreover, the β-1,3-glucanase gene increased in results related to the shoot, which was previously demonstrated to be crucial for flax resistance to *Fusarium* spp.^25^. Flax plants with β-1,3-glucanase overexpression, generating pectinase inhibitors, and employing chitinases and peroxidases increased the production of SMs and changed cell wall compositions, leading to the construction a barrier to fungal growth^26^.

Biological processes, including secondary metabolism, stress, development, cell wall, lipid metabolism, and protein degradation, were commonly altered by all stresses. Such common regulation reflects the flexibility of biological systems through the adjustment of complex metabolism networks in response to stimulants during the evolution of the plant's immobile life^11^.

The stress-responsive common GO terms consisted of AP2/ERF, HB-7, and NAC transcription factor families that significantly upregulated in ABA treatment and drought stress and downregulated in *Fusarium* treatment and K^+^ deficiency. The study of individual stress has identified 11 top TFs, including bHLH (Basic helix-loop-helix), C2H2, NAC, MYB, ERF, bZIP, WRKY, MYB, DREB, HSF, and NFYA10 as known main regulators of abiotic resistance pathways under repeated drought in flax^10^. NAC TFs also contributed to stress response to maize, rice, and flax tolerance to aluminum stress. These TFs, along with MADS-box, adjust plant growth and development and involve cell wall alteration leading to tolerance to aluminum^13^. Most TFs related to potassium starvation have been demonstrated to belong to MYB, bHLH, NAC, B3, bZIP, WRKY, and AP2/ERF, participating in physiological plant processes, stress resistance, and secondary metabolism^18^. The stress signaling pathways share common components comprising ROS, calcium ions, hormones, TFs, and mitogen-activated protein kinase (MAPK) cascades^27^.

The root GO terms showed that biological processes, namely TCA/org transformation, oxidoreductase activity, cell wall, transport, hormone metabolism, and receptor kinase signaling, are specifically modulated under abiotic stress. In a study of flax under individual stress, GO terms of oxidoreductase activity, particularly peroxidases, cell wall, ion homeostasis, and stress response were most changed under unfavorable conditions of the pH and the Zn deficiency^12^.

Some of our results agreed with the individual studies, while some opposed them. These individual stressors might cause different adjustment responses, which comprise various or shared components in plants. When plants simultaneously encounter stress combinations, it could require similar or opposing molecular, physiological, and metabolic responses. The precise choice of which adjustment strategy during multiple stress is presumably to be affected by factors like the intensity of each individual stress, the time course of stress, plant age, and genotype (tolerant or susceptible to any one of the individual stress)^28^. The type of adjustment can influence the accumulation of SMs, causing spatial and temporal modulation of the biosynthetic pathways, improving the chance of survival under long-term stressful environments^29^.

MapMan analysis categorized SMs into 16 groups. Phenylpropanoid and flavonoid pathways were highly affected by all of the stresses. The highest transcript abundance was related to flavonoid and phenylpropanoid pathways under drought stress. The increase of these compounds was extremely related to the balance of carbohydrates between sources and sinks. Also, it has been reported that water potentials were decreased in the plant under severe drought stress, leading to the transport of soluble sugars. Therefore the accumulation of flavonoids and phenolics increased^30,31^. Outcomes related to drought stress in this work showed a reduced five-fold (− 5) transcript abundance in the glucosinolate pathway. According to these results, drought stress significantly reduced glucosinolate content in *Boechera holboellii* Hornem. Á.Löve & D.Löve and some *Brassica carinata* A.Braun cultivars, whereas some *B. carinata* cultivars showed a significant increase in glucosinolate^32,33^. Treatment of potassium sulfate declined glucosinolate content during drought conditions on canola compared to the untreated plants^34^. Roots showed a few changes in the transcript abundance of SMs. However, stress-responsive transcripts are mostly altered under adverse conditions. SMs, such as sinapic acid, lignin, and flavanols, with defensive roles, increased in *Sinopodophyllum hexandrum* Royle under water deficit^35^.

Several phenolic compounds including flavonoids, monolignols, lignans, lignins, coumarins, and hydrolysable tannins, are formed through the phenylpropanoid pathway^36^. The *Linum* genus, particularly *L. album*, contains the highest levels of lignans, especially PTOX. However, many studies have investigated the lignan biosynthesis pathway, the lignan pathway until the end product PTOX has not been completely clarified^8,20^.

This pathway starts with the deamination of phenylalanine and synthesizes 4-coumaroyl-CoA by 4CL. Then, 4-coumaroyl-CoA is converted to caffeoyl-CoA through several reactions by HCT, which catalyzes two different steps, followed by methylation via CCoAOMT and synthesizes feruloyl-CoA^1,37^. Feruloyl-CoA is converted to coniferyl alcohol through two reduction reactions by CCR and CAD^8,38^. Since coniferyl alcohol has been known as a critical precursor in the biosynthesis of PTOX, the above-stated steps were considered upstream^39^. In the present study, CAD contains the highest number of transcripts in the upstream steps showing spatial expression patterns and might have different functional roles in specific organs. For example, *OsCCR10* involves in response to drought in rice root^40^. Knockout of *OsCCR10* with the CRISPR/Cas9 system revealed that drought tolerance reduced rice due to a decline in lignin content in the root^41^. After coniferyl alcohol, the later specified steps of PTOX and its derivative biosynthesis were considered as a downstream. These steps begin with coupling two molecules of coniferyl alcohol to get pinoresinol enantiomers by an oxidase (LAC11) or peroxidase (POD) with the aid of dirigent proteins, depending on plant species^42–44^. *L. usitatissimum* generates both enantiomers (−)- and ( +)-pinoresinol, followed by the stepwise reductive conversion to lariciresinol and then SECO through PLR (1–4)^45^. (−) SECO, resulting from ( +)-pinoresinol, is catalyzed by the action of SDH to matairesinol. Subsequently, matairesinol is converted to deoxypodophyllotoxin by several enzymatic reactions in *P. hexandrum*, such as PhOMT3, CYP71CU1, PhOMT1, and 2-ODD. However, the genes encoding enzymes associated with steps between matairesinol to deoxypodophyllotoxin are not yet identified in *Linum*^8,46,47^. This study realized some of these genes. The transcriptomic analysis of *L. usitatissimum* revealed that the highest expression levels of *LAC11*, *POD*, *4CL*, and *SDH* genes were under drought stress. The roots subjected to abiotic stress demonstrated the downregulation of the expression level of these enzymes. Conversely, quantitative expression of *SDH* using qRT-PCR showed an increasing trend in *L. album* roots and differed in its shoots under all treatment. Furthermore, *2-ODD,* excluding drought stress, and *OMT1* showed a rising trend in *L. album* under all stress, conforming to previously reported studies in *P. hexandrum.* However this pathway evolved independently in the two species^20^. Quantitative expression of genes related to PTOX biosynthesis, studied in different organs of *P. hexandrum*, exhibited that *SDH*, *CAD*, *CCR,* and *cinnamate 4-hydroxylase* genes increased in rhizomes more than roots^37^. The increased gene expressions related to growth and development and PTOX biosynthesis were also reported at 15 °C in *S. hexandrum*. While gene expressions and content of PTOX decreased and genes responsive to stress dominated at 25 °C in this plant^1^. In the study conducted by Kumari et al. (2022), genes of phenylalanine ammonia-lyase (*ShPAL*), *Sh4CL*, *ShC3H*, *ShCCoAOMT*, *ShCOMT*, *ShCAD*, *ShDPO*, *ShPLR*, and *ShSDH* upregulated, as well as, increased PTOX content in root under drought stress. However, there was no evidence of PTOX in the leaf^35^.

High-performance liquid chromatography (HPLC) analysis, consistent with qRT-PCR results, demonstrated an increase of SECO content and upregulation of three selected genes (*SDH*, *OMT1*, and *2-ODD*) led to producing the highest PTOX in the roots after 48 h combination treatment. At the same time, shoots produced PTOX by consuming the precursor of SECO. Under drought stress in the root at 12 h, a copious amount of SECO and overexpression of *2-ODD* caused the conversion of 6-MPTOX and PTOX during stepwise reactions^1^. However, PTOX content declined because of the downregulation of *2-ODD* after 48 h, according to qRT-PCR. Other enzymes related to lignan biosynthesis are probability involved in the 6-MPTOX generation, so PTOX decreased under drought stress. Despite the high SECO amount and overexpression of *2-ODD* (in the shoot after 48 h), PTOX significantly decreased because of the downregulation of *SDH* and *OMT1*. The K^+^ deficiency and drought stress often had opposite accumulation patterns of three selected lignans. The different responses to individual and combined stresses in *L. album* are suggested the complex regulatory mechanism of lignans biosynthesis, which requires further investigation. The expression of *PLR* in *S. hexandrum* contradicts the PTOX content in the different organs, proposing that the PTOX-producing tissue cannot necessarily be its pool^48^.

Application of abiotic and biotic elicitors, including chitosan, methyl jasmonate, salicylic acid, yeast extract, and Ag^+^, has mostly established that the expression of genes associated with lignan biosynthesis and lignan content enhanced in *Linum* spp. in vitro^49–52^. Also, a study conducted on different accessions of *L. album* under drought stress presented different patterns based on physiological and biochemical responses^53^.

Generally, the results from qRT-PCR and HPLC analysis with transcriptomic analysis are consistent, corroborating previous studies. Although they may be different in some cases due to species or genotype differences, type of culture, and stress intensity. The K^+^ deficiency and drought stress often had opposite up/down-regulation patterns, with the negative synergistic effects putting a lot of expenses on the plant. While fusarium's up/down-regulation pattern was similar to K^+^ deficiency, drought stress was similar to ABA treatment.

Therefore, strategies based on transcriptome for species that likely accumulate lignans would aid in identifying common features between species and environmental cues to clarify the PTOX biosynthesis pathway.

## Conclusion

This study has evaluated the common core of gene expression changes using comprehensive transcriptome data of the flax. The results showed that the *EP3* gene increased in all stresses. In analysis of SMs pathways, the most alterations were observed in the biosynthetic pathways of flavonoids and phenylpropanoids. In the phenylpropanoid biosynthesis pathway, out of 12 genes encoding enzymes of the PTOX biosynthetic pathway had the highest expression increase for *LAC11*, *POD*, and *4CL*. The results of HPLC and qRT-PCR were consistent with the in silico results, and all revealed that K^+^ deficiency and drought had opposite patterns. This work suggests new insights into the response of *Linum* species to abiotic stresses that can be employed to improve PTOX for commercial goals.

## Methods

### RNA-Seq data collection and analysis

The public available RNA-Seq datasets for six stress treatments in *L. usitatissimum* were obtained from NCBI GEO (www.ncbi.nlm.nih.gov/geo). The datasets were related to whole-plant, leaf and root organs at the seedling stage (Table 1). Adapter sequences and low-quality reads were trimmed using Trimmomatic v0.39 based on its adaptive trimming algorithm and maximum information (MAXINFO) to balance the benefits of retaining longer reads against the drawback of having low-quality bases^54^. The other trimming criteria were TRAILING: 20, MAXINFO: 80: 0.8, MINLEN: 80, changed based on the length of the reads. The clean reads were mapped to the *L. usitatissimum* reference genome (https://phytozome.jgi.doe.gov/pz/portal.html#!info?alias=Org_Lusitatissimum) using HISAT v2-2.1.0, and the number of mapped reads was counted using python script HTSeq-count v0.12.4 according to GTF file (phytozome-next.jgi.doe.gov/info/Lusitatissimum_v1_0)^55,56^. A gene expression matrix was created and subjected to DEG analysis for each dataset. The DESeq2 R package was applied to identify DEGs. Also, DEGs were found using the edgeR R package for the datasets without any biological replications^57,58^. These packages provide statistical routines for determining differentially expressed genes using a model based on the negative binomial distribution. Up and down-regulated genes were considered differentially expressed if the FDR-corrected *p*-values were ≤ 0.05.

### Functional enrichment analysis

DEGs were subjected to further functional analysis. They were mapped to each functional category with the GO database. Then, a Venn diagram was conducted to identify common significant DEGs of each stress type using Venny 2.1.0 (http://bioinfogp.cnb.csic.es/tools/venny/index.html). In other words, for each stress type, common DEGs among the different datasets were defined as the final DEG lists and were considered for further downstream analysis.

TAIR (The Arabidopsis Information Resource) IDs related to the common DEGs were subjected to GO enrichment analysis using AgriGO^59^. The common DEGs identified across the analyzed studies were functionally classified in the MapMan category. MapMan analysis was primarily performed upon TAIR gene listing by the *A. thaliana* genome mapping files to classify functional categories^60^. The second MapMan analysis gave an overview of gene expression data onto diagrams of the main pathways of SMs biosynthesis for all the transcriptomic datasets individually.

### PTOX biosynthetic pathway genes and PPI network analysis

Annotation and enrichment test of the transcriptome data of *L. usitatissimum* for pathways of PTOX was performed using the Kyoto Encyclopedia of Genes and Genomes (KEGG, www.kegg.jp/kegg/kegg1.html) and Phytozome database^61^. The log2 (fold-change) values of DEGs of each dataset were used to plot the heatmap in R (version 3.5.0). The log2 (fold-change) values of enzyme-related DEGs of the lignan biosynthesis pathway were considered if the FDR-corrected *p*-values were ≤ 0.05, at least in one of the six stresses. The Search Tool for the Retrieval of Interacting Genes/Proteins (STRING) database (http://string-db.org/cgi/input.pl) was used to identify known PPI among the enzyme-related DEGs of the lignan biosynthesis pathway^62^. Networks were visualized by Cytoscape software^63^.

### Plant materials

The plant materials were collected from wild habitats at Imam Khomeini International University, Qazvin, Iran (36.15°N, 117.15°E), then identified by Dr. Ahmadreza Mehrabian (Department of Plant Sciences and Biotechnology, Shahid Beheshti University). A voucher specimen has been deposited in the Herbarium of Shahid Beheshti University (HSBU-2019980).

The plant seeds were sown after germination in 10 cm diameter plastic pots filled with a mixture of peat moss and perlite in a 1:1 ratio under the natural light condition in the greenhouse. Seedlings were then irrigated with Hoagland’s nutrient solution (KH_2_PO_4_, KNO_3_, Ca(NO_3_)_2_ 4H_2_O, MgSO_4_.7H_2_O, H_3_BO_3_, MnSO_4_ H_2_O, ZnSO_4_·7H_2_O, CuSO_4_·5H_2_O, NaMoO_4_·2H_2_O, Na-EDTA, FeSO_4_ 7H_2_O)^64^. Seedlings with three replications were treated with Hoagland’s nutrient solution containing − 9 bar of PEG-6000, Hoagland’s nutrient solution without potassium, and a combination of both treatments^18^. Untreated seedlings were used as controls. The shoot and root organs harvested after 12 and 48 h treatment, separately. The samples were instantly frozen in liquid nitrogen before storing at ‒80 °C. Plant study complies with relevant institutional, national, and international guidelines and legislation.

### qRT-PCR assay

Total RNA was isolated from shoots and roots of *L. album* during the vegetative stage using a column RNA Isolation Kit (DENA Zist, Asia) following the manufacturer’s instructions. The extracted RNA was treated with DNase I (Sigma, USA), and the synthesis of the first strand of cDNA was then performed using Easy cDNA Synthesis (ParsTous, Iran). This experiment was conducted in a completely randomized design with three biological replications and two technical replications. Primers of candidate genes were designed by OLIGOTECH software (OligoTech. Inc., Wilsonville, OR, USA) and Vector NTI software package (Invitrogen, Carlsbad, CA, USA). The qRT-PCR was performed using RB SYBR qPCR Kit (RNAbiotech, Iran). PCR amplification was conducted at 95 °C for 1 min, followed by 40 cycles of desired Tm (See additional file) for 1 min and 72 °C for 15 s. The relative expression of each gene of interest was calculated with REST using the formula 2^−△△Ct^ and expressed relative to GAPDH as the internal control.

### HPLC analysis

The shoot and root samples of *L. album* (1 g) were prepared using a pestle and mortar in liquid nitrogen, followed by extraction in methanol (80% v/v). The samples were then sonicated at room temperature for 1 h. Dichloromethane (4.0 mL) and water (4.0 mL) were added to obtain a partition of compounds between two layers, followed by centrifugation at 5000 rpm for 15 min. The dichloromethane fractions were then collected, dried, and dissolved in 1.0 mL of HPLC-grade methanol for HPLC analysis^65^.

The Lignan content was determined using a Waters liquid chromatography system consisting of a 2695 Separations Module (USA) and a 2487 Dual Absorbance Detector (USA). Data acquisition and integration were carried out with Millennium32 software. The chromatographic separations were performed on a 250 × 4.6 mm Eurospher 100–5 C18 column (KNAUER company, Berline, Germany) with a reversed-phase matrix in a gradient system using acetonitrile (solvent A) and distilled water (solvent B) with a 1 mL min^−1^ flow rate. A UV detector was set at 280 nm. The presence of PTOX, 6-MPTOX, and SECO was identified based on retention time and comparison of UV spectra with the authentic PTOX, 6-MPTOX, and SECO standards purchased from Sigma-Aldrich (Taufkirchen, Germany). Different concentrations of the three compounds (25, 50, 75, and 100 µg mL^−1^) were used for the calibration curves.

### Statistical analysis

One-way ANOVA analysis was conducted using R statistical software v.3.6.0 (freely available at http://www.r-project.org), followed by Duncan’s multiple comparisons at a significance threshold of 0.05. Data were presented as means ± standard deviations (SD).

## Supplementary Information

Supplementary Information 1.

Supplementary Information 2.

## Data Availability

All raw sequence data used to identify genes involved in the PTOX biosynthesis pathway for *Linum* is accessible from NCBI, the National Center for Biotechnology Information, as described in Table 1. Files are available for download through the NCBI website (https://www.ncbi.nlm.nih.gov/bioproject/) with accession numbers PRJNA598287, PRJNA515812, PRJNA414507, PRJNA504749, PRJNA343117, and PRJNA497472.
